# Dental Anatomy

**Published:** 1911-01-15

**Authors:** N. S. Hoff


### DENTAL ANATOMY.

By N. S. Hoff, D.D.S.

In order that a fundamental basis for the study of oral hygiene and dental prophylaxis may be secured it is essential that a clearer statement of the anatomic conditions should be made. It is also clearly evident that this statement will be much more valuable if accompanied with such embryologic and histologic details as are available, and especially where departures from normal physiologic functions produce conditions leading to probable pathologic conditions.

**The Mandible.** This bone forms the base of the face and to it are attached the structures which form the floor and walls of the oral cavity. Its greatest function is to support the sixteen permanent teeth which perform the active function of food mastication. It also furnishes attachment to some of the muscles of the organs of speech and deglutition, and to those which complete the esthetic harmony in facial expression. This is one of the first bones to begin ossification; and its general form is fairly complete at the end of the fourth month of foetal life. At birth the two halves are not yet united at the symphysis and many changes take place in the way of consolidation, interstitial growth and characteristic form as the teeth develop and the muscles begin their functions. The general form is first outlined by the so-called Meckel's Cartilages which are gradually displaced with bone, while the bone itself is developed within a membrane enclosing the cartilage. At birth the places for the first teeth are clearly indicated and the tooth forming organ is seen inclosed in a bony crypt or cell which afterwards becomes the tooth socket.

**The Maxilla.** The upper jaw bone consists of two bones, which, although closely adapted in a median suture extending mesio-distally in the roof of the mouth, which never becomes solidly ossified. In the early embryonic period there is also another suture between the canine and lateral teeth separating a portion of the maxilla which eventually contains the four incisor teeth and which is called the pre-maxilla. Failure to close either of these sutures by normal development results in so called cleft palate deformities, often so extended as to produce double cleft in the lip, (hare lip) and a more or less wide splitting of the soft palate and uvula, called staphyloschisis.

The maxilla begins to ossify at nearly as early a period as the mandible. It has no cartilaginous pre-formation, but is formed intramembraneous from six centers of ossification. The bone has thin walls everywhere except the alveolar portion containing the roots of the teeth. It contains a large air cavity, the antrum, and with other bones forms the nasal air passages, and the hard palate or the roof of the mouth. At birth its longest diameter is its antero-posterior measurement, but as the alveolar process develops to support the roots of the teeth, and the antrum cavity enlarges its perpendicular measurements is longest.

**Bone Calcification.** Bone is formed by special cells called bone corpuscles which secrete calcium salts by four methods into various forms of matrices of soft tissue. *Interstitial calcification* results when the lime is deposited in the embryonic connective tissue. *Intra-membraneous calcification* is illustrated in the thin flat bones, as in the maxillae, where an organized membrane supplies the matrix. *Sub-periosteal calcification* is seen as the result of deposits from the bone surface of the periosteal covering of all bones, as in the cortical surface of the long bones. *Intra-cartilaginous calcification* is the transformation of the embryonic cartilaginous structures, as the head of the femur or body of the mandible, into bony structures. The cells which deposit bone material are called *osteoblasts*, and perform their function in a similar way regardless of the particular environment or class of calcification. They do not calcify internally, but secrete the calcic salt into the particular form of matrix and recede until their work is accomplished. In different classes of bones we have characteristic bone formations. In the long bones the deposition is in concentric circles inclosing a nutrient canal, these circles become coalesced in various forms of bones. For the ultimate nutrition of the bone each Haversian circle contains small spaces or lacuna filled with lymph plasma, from which radiate smaller canals or canaliculae which anastomose with other canaliculae forming a complete nutrient system. In subperiosteal calcification the large Haversian canals are absent, but in the cementum of the tooth root we sometimes find canals similar to those seen in larger bones. The mandible is calcified in large part interstitially, and a considerable part of the maxilla is also calcified in the same way. This is made necessary because of the later development of the teeth and the alveolar process supporting their roots. The crowns of the teeth are at first encysted in their bony crypts which are absorbed as the roots develop and the crowns emerge from the bone. In doing so the bone forming osteoblasts are carried upward and build in around the tooth root the supporting alveolus. This absorption and rebuilding process is of great interest and importance in connection with all prophylaxis endeavors to secure physiological eruption of the deciduous teeth, and the prevention of malocclusion of the permanent teeth. It also needs more study with reference to the rescue treatment of destructive peridental diseases which prevail largely in patients past the formative periods in life, or when the degenerative processes are more active than the reparative.

**Development of the Dental Structures.** We find it quite difficult to make a statement of the chronological development of the dental organs as no one has attempted so extensive a research as this would involve; but it seems so essential as a basis for an intelligent ante-natal prophylaxis that we will make the best statement obtainable from various literary sources at our command. The statement made by H. W. Phaff of the findings of Carl Rose, from a research on the human embryo, is perhaps as reliable as can be found and will guide us largely in this work. At the tenth week the human embryo is about one inch in length and weighs about one dram. At this period the *Dental Ridge* is distinctly seen. It has begun to show indications as to the positions of the tooth follicles by enlargements at the points where the future teeth are to develop. These enlargements soon take on a club shape, and the dental papilla consisting of a collection of mesodermal cells seem to be forcing it way up under the ridge making a cap form of the outer cells. At fourteen weeks the foetus has grown to three or four inches and weighs five or six drams. At this time the temporary tooth germs are beginning to separate from the dental ridge to become distinctly differentiated cells, the stellate reticulum appears and the distal end of the ridge dips down and disappears into the mesoderm having lost its connection with the epithelium. At the seventeenth or eighteenth week the epithelial bands attaching the anterior teeth to the ridge are exceedingly attenuated while those for the posterior teeth remain broad. The dental papilla for the first permanent molar is noted at this time. At twenty weeks the foetus has grown to four or five inches and weighs two or three ounces. The first traces of calcification of dentine is observed taking place on the tooth follicles for the incisor teeth. and the follicle of the first permanent molar is outlined although calcification of this tooth does not begin until considerably later.

At six months the foetus weighs from six to eight ounces and calcification of the deciduous tooth crowns are well under way and the dental ridge is being rapidly absorbed. At eight months the foetus weighs about two pounds and the cusps of the molar teeth are well formed with both dentine and enamel being deposited, but a complete cap has not yet formed. The dental follicles for the permanent incisors are forming and there is a thickening of the dental ridge in the region of the premolars. At the thirty-fourth week the foetus has increased in size and weight to about four pounds and the caps for the crowns of the deciduous teeth are well formed, but no trace of calcification is noted in the first permanent molar, its germ seems to lie dormant for several years. At birth the child has increased in size and weight, very much more rapidly than the teeth have developed. There is a great variation in the size and weight of the newly born baby and it varies from four to seven or eigth pounds. There is a case on record of a very large woman who was delivered of a twenty-three pound babe. There are also cases on record of children being born with one or more fully developed and erupted teeth, but no such condition as a fully developed tooth or crown is usual at this period, although in many cases the coronal portion of the tooth is well calcified, anf the calcification of the first permanent molars are well started.

In order that we may more clearly present the process of development in the various dental tissues we shall briefly sketch each of these different organs and tissues.

**The Tooth Follicle.** About the twelfth week of foetal life the tooth germ, made up of enamel and dentine organs, becomes separated from the tooth-band and is inclosed in a sack-like covering of periosteal tissue which eventually forms the alveolo-dental membrane and from it the cementum of the root is deposited; this constitutes the dental follicle. As the alveolar process and tooth root develop this follicular wall gradually becomes changed into the pericementum or connecting ligament attaching the tooth to the alveolar process.

**Peridental Membrane.** This is a strong fibrous membrane. lining the root socket and serving as the medium connecting the tooth root and alveolus. It is made up mostly of bundles of inelastic connective tissue fibers, which pass from the root to the alveolus mainly in a horizontal direction, except that the individual bundles diverge circumferentially in antagonistic directions, preventing the rotation of single rooted teeth in their sockets. Toward the root apex the fibers assume a vertical or axial direction. At the gum margins the fibers disappear in the submucosa of the gum tissue and the alveolar periosteum. At the root apex the tissue is much thicker and the connective tissue less dense, and it is richly supplied with blood vessels and nerve trunks which pass into the periosteum and pulp. The blending of the peridental membrane and pulp tissue is very close and without special difference in structure, until the apical foramina is passed. On the root side the membrane is securely attached by its fibers in the lamella and pitted surface of the cementum; while on the alveolar side it is attached in the crypts and rough bony surface of the alveolus. Between the bundles of fibers, especially toward the gum margins are found masses of epithelial cells, by some supposed to be remnants of the enamel organ, and by other specialized glands; but as no one has described a function for these glands their existence is a matter of further research. Blood vessels and nerves are abundantly distributed throughout the membrane, having their source largely from the apical region, but greatly supplemented by anastamosing vessels from the gum tissue and alveolus.

The peridental membrane may be properly classified as an organized structure. Its function is to first complete the root calcification by forming the cementum; and its second function is to retain the tooth in its place in the jaw, and in a large measure to provide the tactile sense to each individual tooth which makes it one of the most important organs in the dental system. Its preservation in a healthful condition constitutes one of the most important aims in the treatment of peridentitis. In its normal state it serves as a most useful accessory organ to the senses of touch; taste (?), and hearing, as it appeals to these senses not only by its direct connection with the sensorium, but by the direct and collateral reflexes as well. The membrane is myxomatous in its developmental stages but becomes fibrous in maturity. Because of its intimate relations with the tooth pulp it is subjected to inflammatory and suppurative diseases of that organ, and because of its exposure at the gingiva it is liable to external infection. It is also occasionally the seat of internal infections and abscesses which are not easily explained or successfully treated. It reacts to auto-infection or general systemic diseases. In old people it is one of the first tissues to show the progress of senile degeneration. In oral prophylaxis it is the most important organ involved, and later on it will require special consideration because of its pathologic and therapeutic bearings.

**The Enamel Organ.** This organ is derived from the outer or epithelial layer of cells and as an organized structure covers the dentinal papilla as a crown or cap. The organ is bell shaped and is made up from two layers of epithelial cells inclosing a pulpy structure, composed of star shaped cells and the stratum intermedium, between which and the dentinal papilla is the structure containing the ameloblasts, or enamel forming cells. Enamel begins to form about the sixth month of foetal life and shortly after the dentine has begun to calcify and a thin cap of dentine has deposited. Bodecker says that the enamel is deposited on the newly formed dentine by the medulary corpuscles which lie nearest to the dentine, and these are derived from the previous ameloblasts. These finely granular medulary corpuscles are infiltrated with lime salts and thus enamel is produced. As an organized tissue it is laid on or becomes attached to the newly formed dentine, which has an irregular surface with bay like indentures.

With a normally developed organ and a good supply of material we should have ideal calcification, and it will be exceedingly difficult to foresee and prevent accidental and unexpected interferences which might deform or interfere with the perfect development of the tooth germ and subsequently with its normal function. But we know from clinical observation that faulty enamel is usually found in that portion of the several teeth where calcification takes place at the same periods, and we must conclude that some aberation has taken place either at the same period in the organs development or at the same period in the calcification of the enamel. We are inclined to think that it must be during the calcification period because we often observe that the dentine is also affected. But there are many cases where the dentine seems well formed under very defective enamel. We also observe that some tooth germs never appear at all, or if so they never calcify, and such phenomena, unlike defective calcification, are usually asymetrical, indicating a local rather than a general or systemic cause. The determination of the cause of these abnormalities becomes an exceedingly interesting problem, made more difficult and intricate because of the great difficulty in securing an acurate history of the mothers physical health during the gestation period. Some unnoticed accident or experience which passed easily from the memory may have occurred that would account for many of the malformations for which we can find no justifiable cause. This ought to become a matter of clinical observation and record. If we could have the co-operation of the physician and nurse, or our patients could be impressed with the importance of the matter we might accumulate data that would give us a better insight into the cause of this important pathologic condition.

